# Repeatability of Feather Mite Prevalence and Intensity in Passerine Birds

**DOI:** 10.1371/journal.pone.0107341

**Published:** 2014-09-12

**Authors:** Javier Diaz-Real, David Serrano, Javier Pérez-Tris, Sofía Fernández-González, Ana Bermejo, Juan A. Calleja, Javier De la Puente, Diana De Palacio, José L. Martínez, Rubén Moreno-Opo, Carlos Ponce, Óscar Frías, José L. Tella, Anders P. Møller, Jordi Figuerola, Péter L. Pap, István Kovács, Csongor I. Vágási, Leandro Meléndez, Guillermo Blanco, Eduardo Aguilera, Juan Carlos Senar, Ismael Galván, Francisco Atiénzar, Emilio Barba, José L. Cantó, Verónica Cortés, Juan S. Monrós, Rubén Piculo, Matthias Vögeli, Antoni Borràs, Carlos Navarro, Alexandre Mestre, Roger Jovani

**Affiliations:** 1 Estación Biológica de Doñana (CSIC), Sevilla, Spain; 2 Departamento de Ecoloxía e Bioloxía Animal. Universidade de Vigo, Campus As Lagoas Marconsende, Vigo, Pontevedra, Spain; 3 Departamento de Zoología y Antropología Física. Universidad Complutense de Madrid, Madrid, Spain; 4 Grupo Ornitológico SEO-Monticola. Unidad de Zoología. Universidad Autónoma de Madrid, Madrid, Spain; 5 Departamento Biología Animal, Vegetal y Ecología, Universidad Autónoma de Barcelona, Bellaterra, Barcelona, Spain; 6 Department of Evolutionary Ecology, Museo Nacional de Ciencias Naturales (CSIC), Jose Gutiérrez Abascal, Madrid, Spain; 7 Laboratoire d'Ecologie, Systématique et Evolution, CNRS UMR 8079, Université Paris-Sud 11, Bâtiment 362, Orsay, France; 8 Evolutionary Ecology Group, Hungarian Department of Biology and Ecology, Babeş-Bolyai University, Cluj Napoca, Romania; 9 MTA-DE “Lendület” Behavioural Ecology Research Group, Department of Evolutionary Zoology and Human Biology, University of Debrecen, Debrecen, Hungary; 10 'Milvus Group' Bird and Nature Protection Association, Târgu Mureş, Romania; 11 Unidad Mixta de Investigacion en Biodiversidad. Instituto Cantábrico de Biodiversidad (CSIC-Universidad de Oviedo), Oviedo, Spain; 12 Unidad Asociada de Ecología Evolutiva y del Comportamiento, Museo de Ciencias Naturales de Barcelona (CSIC), Barcelona, Spain; 13 Cavanilles Institute of Biodiversity and Evolutionary Ecology, University of Valencia, Paterna, Spain; 14 Parque Natural del Carrascal de la Font Roja, Alcoi, Spain; 15 Federal Office for the Environment FOEN, Species, Ecosystems, Landscape Division, Bern, Switzerland; 16 Profesor Gonzalo Sanchez Vazquez, Sevilla, Spain; 17 Department of Microbiology and Ecology, University of Valencia, Burjassot, Spain; Hungarian Academy of Sciences, Hungary

## Abstract

Understanding why host species differ so much in symbiont loads and how this depends on ecological host and symbiont traits is a major issue in the ecology of symbiosis. A first step in this inquiry is to know whether observed differences among host species are species-specific traits or more related with host-symbiont environmental conditions. Here we analysed the repeatability (R) of the intensity and the prevalence of feather mites to partition within- and among-host species variance components. We compiled the largest dataset so far available: 119 Paleartic passerine bird species, 75,944 individual birds, ca. 1.8 million mites, seven countries, 23 study years. Several analyses and approaches were made to estimate R and adjusted repeatability (R_adj_) after controlling for potential confounding factors (breeding period, weather, habitat, spatial autocorrelation and researcher identity). The prevalence of feather mites was moderately repeatable (R = 0.26–0.53; R_adj_ = 0.32–0.57); smaller values were found for intensity (R = 0.19–0.30; R_adj_ = 0.18–0.30). These moderate repeatabilities show that prevalence and intensity of feather mites differ among species, but also that the high variation within species leads to considerable overlap among bird species. Differences in the prevalence and intensity of feather mites within bird species were small among habitats, suggesting that local factors are playing a secondary role. However, effects of local climatic conditions were partially observed for intensity.

## Introduction

Why some organisms are abundant while others are rare and how large populations can grow are major questions in ecology. Symbiosis is the most abundant life style in nature [1], although the above questions are still poorly understood in the host-symbiont context. Host-symbiont systems represent an interesting study subject because two complementary approaches exist to explain commonness and rarity: either from the point of view of the host (i.e. why some host species harbour more symbionts than others) or the symbiont (i.e. why some symbiont species are more abundant than others). Under these two approaches much has been done to understand how species-specific and environmental factors affect symbiont abundance. Typically, comparative studies from the host's point of view have attempted to understand which species-specific traits of hosts (e.g. body size, food resources for symbionts) shape the abundance of symbionts (e.g. [2], [3]). Host-focused intraspecific studies have shown how host individual features (e.g. age, sex, body condition) and environmental variables (e.g. weather, abundance of symbiont vectors) shape the abundance of symbionts among host individuals or populations within host species (e.g. [4]–[7]). The same approach has been used from the symbionts' point of view [8], [9]. Note that in case of this approximation the symbiont's environment, especially for ectosymbionts, is not only the host itself but the host's habitat as well.

Overall, there is an unresolved conflict between studies showing a huge (environmentally-governed) variability of symbionts within host species (i.e. between individuals or populations), and studies showing that there are species-specific features that can explain either why some host species have a higher abundance of symbionts than others, or why some symbionts are more abundant than others irrespective of the inhabited host. Surprisingly, little has been done to solve this apparent contradiction between the two approaches, by analysing the relative importance of species-specific vs. local environmental variables to understand why some symbiont species are common while others are rare in host-symbiont systems [10].

As far as we know, few studies have analysed (from the symbiont species or community point of view) symbiont abundance and prevalence repeatabilities when symbionts are occurring on different host species. Previous findings showed that the abundance and the intensity of infection are more repeatable than the prevalence [10]–[16]. Only two previous studies (to our knowledge) have focused on the repeatability of abundance, prevalence, and intensity of infection or richness of symbionts among host species (from the host point of view). They found that abundance and the prevalence of parasites were repeatable among host species, while the repeatability of prevalence was weaker than for abundance [17], [18]. Moreover, a few comparative tests also showed that the studied variable was repeatable at the host species level before using that variable in comparative analyses (e.g. [15], [19]). Therefore, current evidence shows that, while environmental factors shape host-symbiont interactions [20], there are species-specific traits of both hosts and symbionts that consistently shape the outcome of the interaction under environmental stochasticity [7]. Thus, this ultimately leads to higher similarity in symbiont population attributes within host species than between host species.

Here we investigate astigmata feather mites that live on the surface of the wing feathers of birds and are the commonest avian ectosymbionts [21]–[23]. The nature of the biological relationship between feather mites and birds is still poorly understood, and empirical studies show a puzzling scenario: some studies have shown that feather mite abundance correlates positively with bird's body condition [24], [25], while others have found no significant correlation suggesting commensalism [26] or negative correlations and experimental evidence suggesting parasitism [27]–[29]. A recent correlative study analysing a large dataset from 83 species has shown a largely positive relationship with host condition though with a small effect size, which suggests a commensal interaction [30] agreeing with previous experimental results [31]. Another recent correlative study found that uropygial secretions and feather mites reduce hatching failure in birds by reducing bacteria loads in eggshells, showing a mutualist relationship [32]. Overall, this suggests a multifaceted complex of interactions resulting in conditional outcomes for both host and symbiont [33].

In this system, there is also an apparent conflict between intraspecific and interspecific comparative studies. The former show important differences between individuals (e.g. by age, sex, body size, body condition, [24]–[26]) and populations (e.g. by migratory status of the population, [34]), or along environmental gradients (e.g. salt concentration in the air, [6]). Multispecies comparisons have shown how mean abundance of feather mites correlate with other species-specific traits of birds such as migratory status, sociality, body size, plumage coloration or size of the uropygial gland [2], [3], [27], [35], [36]. However, it has never been tested whether feather mite intensity and prevalence could be considered a species-specific trait in birds. If abiotic environmental variables (but not bird species) were the main determinants of differences among bird species, previous studies would be (at least partially) indicating a hidden correlation between species features and the habitats where these bird species live.

To do so, we analysed for the first time repeatability of intensity and prevalence of feather mites on the flight feathers of birds using the largest dataset to date, which comprised 119 species of passerine birds occurring in distant localities from seven North African, European and Asian countries in the Northern hemisphere. We tested for the repeatability of intensity and prevalence of feather mites because they have different meanings and ecological implications. We also controlled for potentially confounding biological and methodological factors that could systematically bias our repeatability estimates.

## Material and Methods

### Ethics statement

The study was conducted under the current laws of the different countries where it was done. All sampling was conducted with permission from the local government at the sampling site and under the appropriate permits when required (refer to Information S1 for the license numbers). No endangered species were involved. All birds were studied with non-invasive methods and released at sampling locality some minutes after capture.

### Dataset

**Figure 1:**
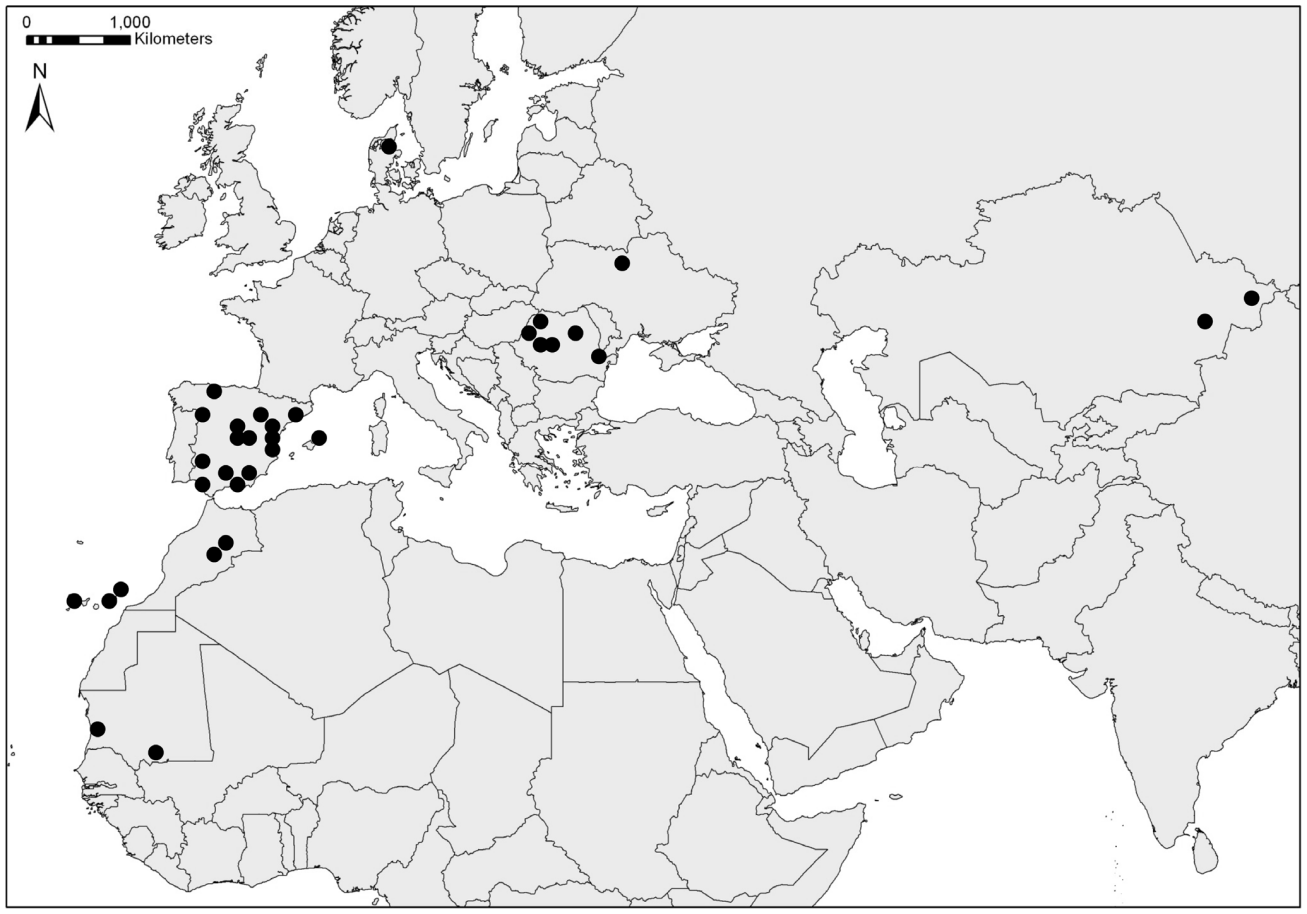
Study area and spatial distribution of data. Close sampling points are summarised by black circles. Countries (sampled bird individuals): Mauritania (85), Morocco (105), Spain (70,321), Denmark (2,394), Romania (1,827), Ukraine (1,175) and Kazakhstan (37).

Data were obtained from “FeatherMites”, a collaborative dataset on feather mite occurrence with data gathered by 89 researchers between 1989 and 2012 from seven countries (Fig. 1). See Information S1 for a summary of the dataset (dataset available under request).Birds were mostly captured using mist nests and kept individually in cloth bags until they were banded, inspected for feather mites and released. Feather mite occurrence was assessed exposing the wing against the sunlight and inspected from the dorsal and ventral surface of each primary, secondary and tertial feathers of one wing (the number of feather mites on both wings of a bird are highly repeatable; [37], [38]). Moreover, for a subsample of birds with at least one feather mite on the wing, the total number of feather mites in one wing was also counted. In 853 individuals (<0.02% of the whole dataset) of Greenfinch (*Carduelis chloris*) the total number of feather mites was not counted, but it was estimated by linear regression from data on the number of primary feathers with more than five mites (R^2^ = 0.48), or from counts of the number of mites on primaries only (R^2^ = 0.87). In all analyses, to avoid pseudo-replication, only the first observation of each individual bird was used in the analyses.

The dataset was analysed separately for the prevalence and the intensity of feather mites. For the analysis of prevalence, we used data on the occurrence of feather mites (whether the individual bird had one or more feather mites on the wing, 1, or not, 0). For the analysis of the intensity of feather mites, we used data of the number of feather mites on the wing of birds with at least one mite. Only bird species with at least 25 individuals were used to avoid model convergence issues during prevalence analyses. After applying these constraints the final sample size for the prevalence dataset comprised 75,944 individuals from 97 species of passerine birds (from 57 genera and 27 families) inspected for feather mites. The intensity database contained 27,457 individuals from 119 species belonging to 62 genera and 28 families. We analysed the distribution of variance in intensity and prevalence across taxonomic levels in a nested analysis of variance for species, genus and family [36], [39]. Species accounted for the highest proportion of total variance (see Results) so repeatability analyses were centred to the species level.

### Statistical analyses

#### Repeatability

Repeatability (the intra-class correlation coefficient) is the proportion of variation that can be attributed to between-group differences [40]; in our case study, between bird species. Here we used this statistic to test whether prevalence and intensity of feather mites could be considered bird species-specific traits; i.e. traits that are more variable between than within bird species.

Repeatability of feather mite prevalence was calculated as R_logitM_ = 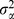/(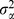
+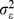), where 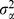 is the between-groups variance (e.g. species) and 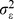 is the residual variance (i.e. between individuals within species). Residual variance was calculated as 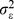 = 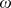 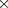
(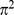/3), where 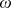 is the multiplicative overdispersion parameter, and 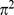/3 is the distribution-specific variance for the logit model. Because prevalence is a proportion variable, these variances were obtained by fitting Generalized Linear Mixed-effects Models (GLMMs) with binomial distribution of errors and logit link function in which bird species identity was included as a random effect. In general, penalized-quasi likelihood (PQL) estimation was used with multiplicative overdispersion [40] except in two analyses, where additive overdispersion was used due to convergence issues. In any case, R values from additive and multiplicative models for binary data have been found to be similar in the presence of overdispersion [40]. We used parametric bootstrapping to obtain 95% confidence intervals (CI; nboot = 1000, npermut = 1000).

Feather mite intensity data were log_10_-transformed and R was calculated as R = 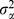/(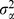
+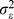). Linear mixed-effects models (LMEs with normal error distribution and identity link function) with bird species identity as a random variable were used to retrieve 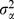 and 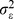. Restricted maximum likelihood (REML) was used for parameter estimation because of its flexibility to control for confounding factors ([40]; see below). Parametric bootstrapping was used, as detailed above, to obtain 95% CI.

#### Adjusted repeatability

Adjusted repeatability (R_adj_) is the repeatability after statistically controlling for confounding effects [40]. Confounding effects are biological and methodological factors that can potentially systematically bias the intensity or prevalence of feather mites, thus artificially either increasing or decreasing repeatability estimates. Thus, R_adj_ is closer to real repeatability after known biases are taken into account. The following confounding factors were considered:

*Observer*. — Some researchers could produce consistently higher feather mite counts or prevalence estimates than others. This, for instance, could lead to higher within-species variance when clumping data from different observers, thus reducing R estimates. Alternatively, species sampled only by one or few observers could misleadingly increase assessed feather mite load differences among species (i.e. increases 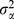); then leading to R overestimation. To correct for this potential observer bias we included observer identity (N = 89) as a random factor (“observer”) in statistical analyses.

*Breeding period*. — Feather mites live permanently on birds, and the main dispersal mode is thought to be from bird to bird when birds are in contact [23]. Thus, the main transmission moment seems to be in the nest from parents to offspring [23]; but also between related and unrelated conspecifics in communal roosts [24], [25]. Therefore, it is not surprising that feather mite intensity and prevalence vary during the year, with birds having the lowest intensities of feather mites during the breeding season when the number of feather mites per bird dilutes, and feather mite populations in each individual have not started to recover [3], [24], [35], [41], [42]. We thus included the categorical variable “breeding period” with two levels, breeding and non-breeding period, as a fixed effect in statistical analyses. The year was divided in two equal periods of six months starting at egg laying based on breeding phenology for each species [43].

*Local climatic conditions*. — As ectosymbionts are ectotherms, feather mites are not only exposed to factors directly governed by the bird host, but also to abiotic environmental factors such as precipitation and temperature. Accordingly, feather mites are known to move within their hosts to meet their favourable conditions [24], [44]. In our study, we analysed the possible differences between localities in terms of environmental factors by two different approaches: weather description and habitat classification. For weather description, we used six different climatic variables: annual mean temperature, mean temperature of the warmest quarter of the year, mean temperature of the coldest quarter, and the same 3 variables for precipitation. Data were obtained from BIOCLIM (http://www.worldclim.org) for each locality. All six variables were highly correlated, and a principal component analysis (PCA) with varimax rotation was used to summarise weather information. The PCA was carried out using “prcomp” function from the “stats” package in R [45] with default settings. The first axis of the PCA (PC1) accounted for 84% of the variance, positioning localities along an axis from dry and warm to wet and cold. Thus, we included the continuous variable “PC1” as a fixed effect in the statistical models.

*Spatial autocorrelation*. — A spatial term of the form *x+y+x^2^+xy+y^2^+x^3^+x^2^y+xy^2^+y^3^* [46] was included to control for spatial autocorrelation as fixed effect in statistical analyses, where *x* and *y* are longitude and latitude coordinates of the sampling sites, respectively. Prior to the analyses, coordinates were centred on their respective means to reduce collinearity with higher order terms [45] and standardized to unit variance.

*Habitat*. — Different habitats could provide different environmental conditions for feather mites thus shaping their populations. The habitat of each study locality was classified as Atlantic forest, crops, Mediterranean and continental forest, river forest, steppe, subalpine meadow, or wetland and included as fixed effect in statistical models.

#### Complementary analyses

To test for consistency in our results, complementary analyses were performed. R and R_adj_ were also obtained separately for the datasets from researchers with at least 25 species sampled at least in two different habitats: five researchers with an N range = 1,256–3,217 birds sampled for intensity and seven researchers with N range = 2,024–3,454 for prevalence. Moreover, given that the habitat consistently entered as confounding factor in the models, we explored whether species had a consistent prevalence and intensity independent of the habitat where they were captured. To do so, we analysed a subsample of ten well-sampled resident bird species (N>25 individuals of each species in each habitat, N = 28,340 birds for prevalence and N = 7,136 for intensity) captured in three habitats (wetland, Mediterranean and continental forest, and river forest).

The rptR package [47] for software R [45] was used to calculate R and its 95% CI for intensity and prevalence. It was also used to calculate R_adj_ of feather mite intensity and its 95% CI. However, binomial errors are not implemented in this package for R_adj_ of prevalence (not elsewhere for R_adj_, H. Schielzeth pers. comm.). Thus, we used SAS 9.2 software (SAS Institute, Cary, NC, USA) for estimating R_adj_ from 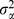 and 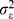 estimates retrieved with SOLUTION statement, and 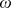 with the RANDOM statement (indicating "_residual_") in the GLIMMIX procedure. Significance of R_adj_ estimates for prevalence was calculated comparing twice the difference in log-likelihoods of models with and without the random effect, against the χ^2^ distribution with one degree of freedom [40]. We used backward stepwise GLMMs and LMEs starting from the saturated model to assess the effect of each of the explanatory variables on prevalence and intensity (variables with p>0.05 were excluded). Thus, only statistical significant variables were used in R_adj_ estimations.

## Results

### Prevalence

**Figure 2:**
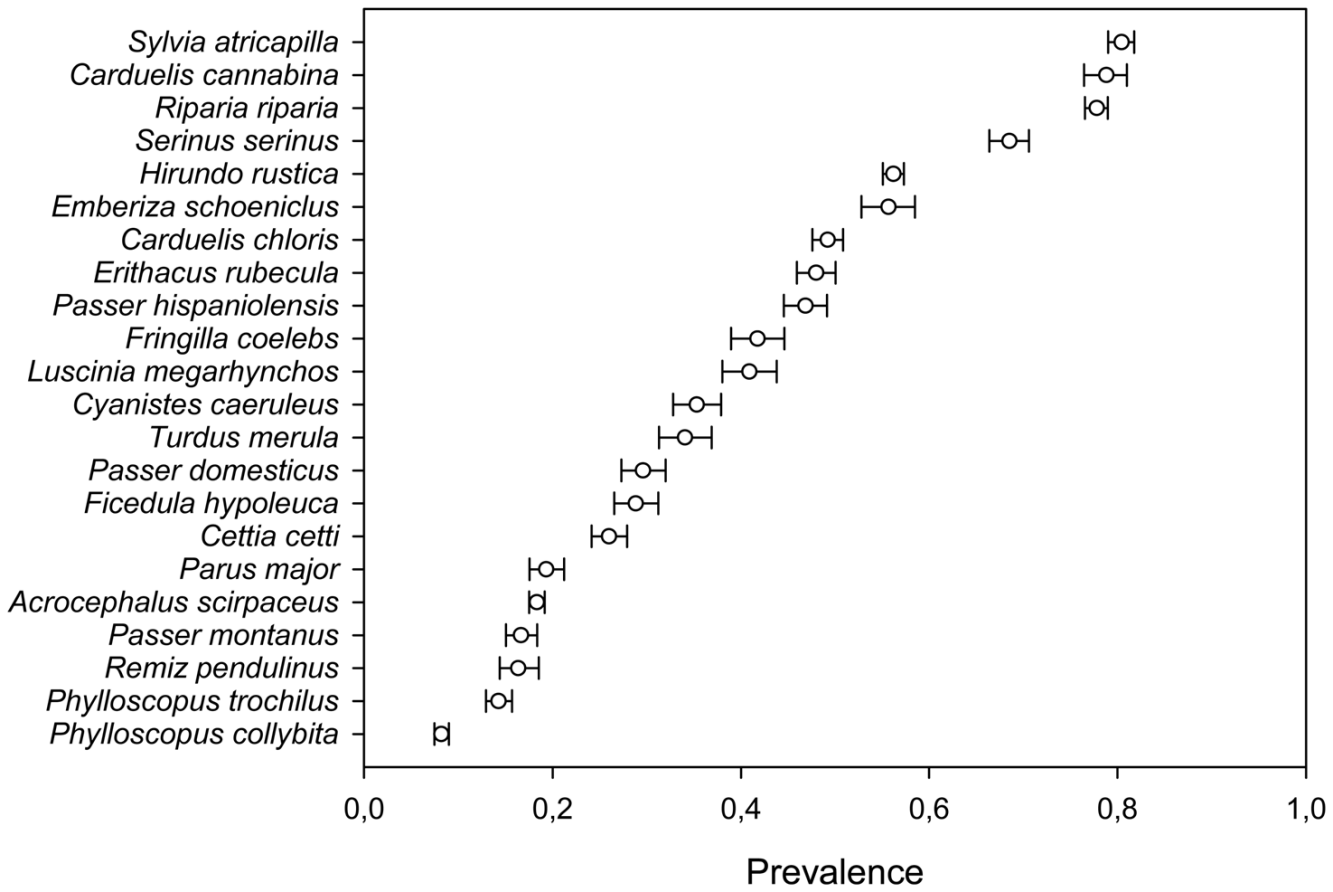
Prevalence of feather mites of birds. Feather mite prevalence (proportion of birds with feather mites) in bird species with data for more than 1,000 individual birds with corresponding 95% confidence interval estimates.

**Figure 3:**
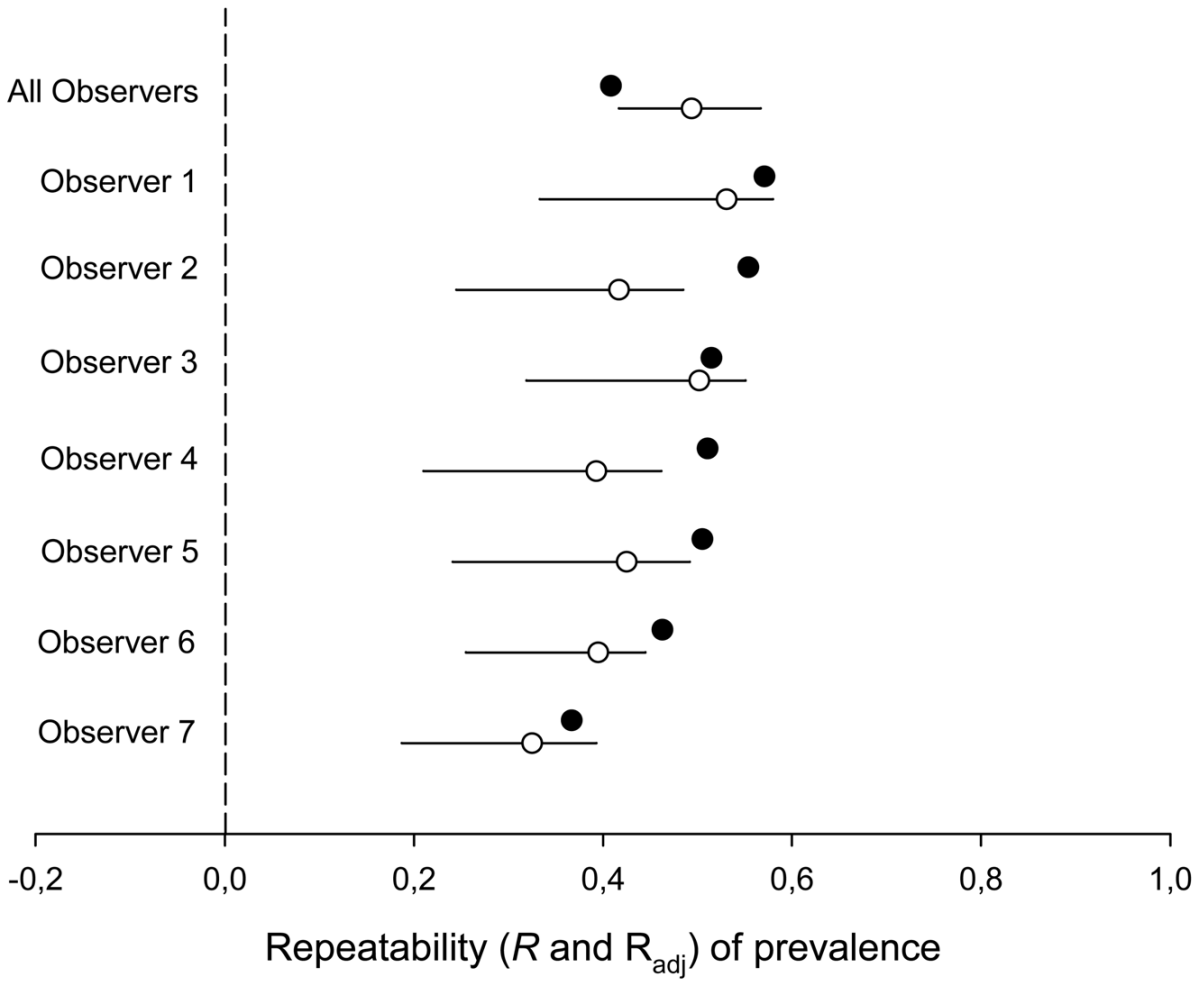
Species repeatability and adjusted repeatability of feather mite prevalence. Species repeatability (R; white circles) and adjusted repeatability (R_adj_; black circles) for feather mite prevalence with corresponding 95% confidence interval estimates for different intensity data subsets (either the entire dataset, “All observers”, or for data from seven different researchers separately). The following confounding effects were retained in the models: “breeding” was retained for observers 1–7; "habitat" was also retained for observers 2, 5 and 6; the “PC1” was also added for observers 1 and 4; finally, six variables of the spatial autocorrelation term were retained for observer 4. For “All Observers” the final model included the fixed effects shown in Table 1. All R and R_adj_ estimates were statistically significant at α = 0.001. 95% CI could not be calculated for R_adj_ (see Methods).

Mite prevalence was very variable among species (Fig. 2), ranging from 0% in *Sylvia hortensis* up to 100% in *Rhodospiza obsoleta* (see Table S1 for detailed results for each species). Analysing the whole dataset we found a moderately high repeatability of prevalence among species (R = 0.494). This result, as well as all other repeatability estimates reported in this study was statistically significant (p<0.001). Prevalence was higher during the non-breeding season, differed among habitats, and showed spatial autocorrelation (Table 1). After controlling for these factors we found an R_adj_ = 0.409, showing that while several variables explained part of the among-species variation, differences in prevalence of feather mites were consistent at the species level (Fig. 3).

**Table 1:** Parameter estimates for GLMM of the dataset of prevalence of feather mites on birds.

Factor	Description	Estimate	SE	df	t	F	P	
Intercept		0.681	0.527	88	1.29		0.1996	
Breeding period				1, 75747		1088.46	<.0001	
	Non breeding	0.916	0.028	75747	32.99		<.0001	
	Breeding	0	.	.	.		.	
Habitat				6, 7532		18.20	<.0001	
	Atlantic forest	−1.583	0.514	75747	−3.08		0.0021	
	Crops	−1.809	0.497	75747	−3.64		0.0003	
	Steppe	−1.450	0.511	75747	−2.84		0.0045	
	Wetland	−1.785	0.499	75747	−3.58		0.0003	
	M. and C. forest	−1.972	0.499	75747	−3.95		<.0001	
	River forest	−1.585	0.499	75747	−3.18		0.0015	
	Sub. meadow	0	.	.	.		.	
Autocorrelation								
	y	0.416	0.052	75747	8.06		<.0001	
	xy	0.658	0.055	75747	11.88		<.0001	
	y^2^	−0.217	0.033	75747	−6.50		<.0001	
	x^3^	0.008	0.001	75747	6.45		<.0001	
	x^2^y	−0.136	0.015	75747	−8.93		<.0001	
							

For continuous variables, only statistically significant variables are shown.

#### Different observers

The same analyses were repeated separately for datasets from the researchers contributing the highest sample sizes. Similar results were found as when analysing the whole dataset (R range: 0.325–0.531; R_adj_ range: 0.367–0.571; see Fig. 3 for the confounding effects retained in the analyses of each subsample).

#### Different habitats

**Figure 4:**
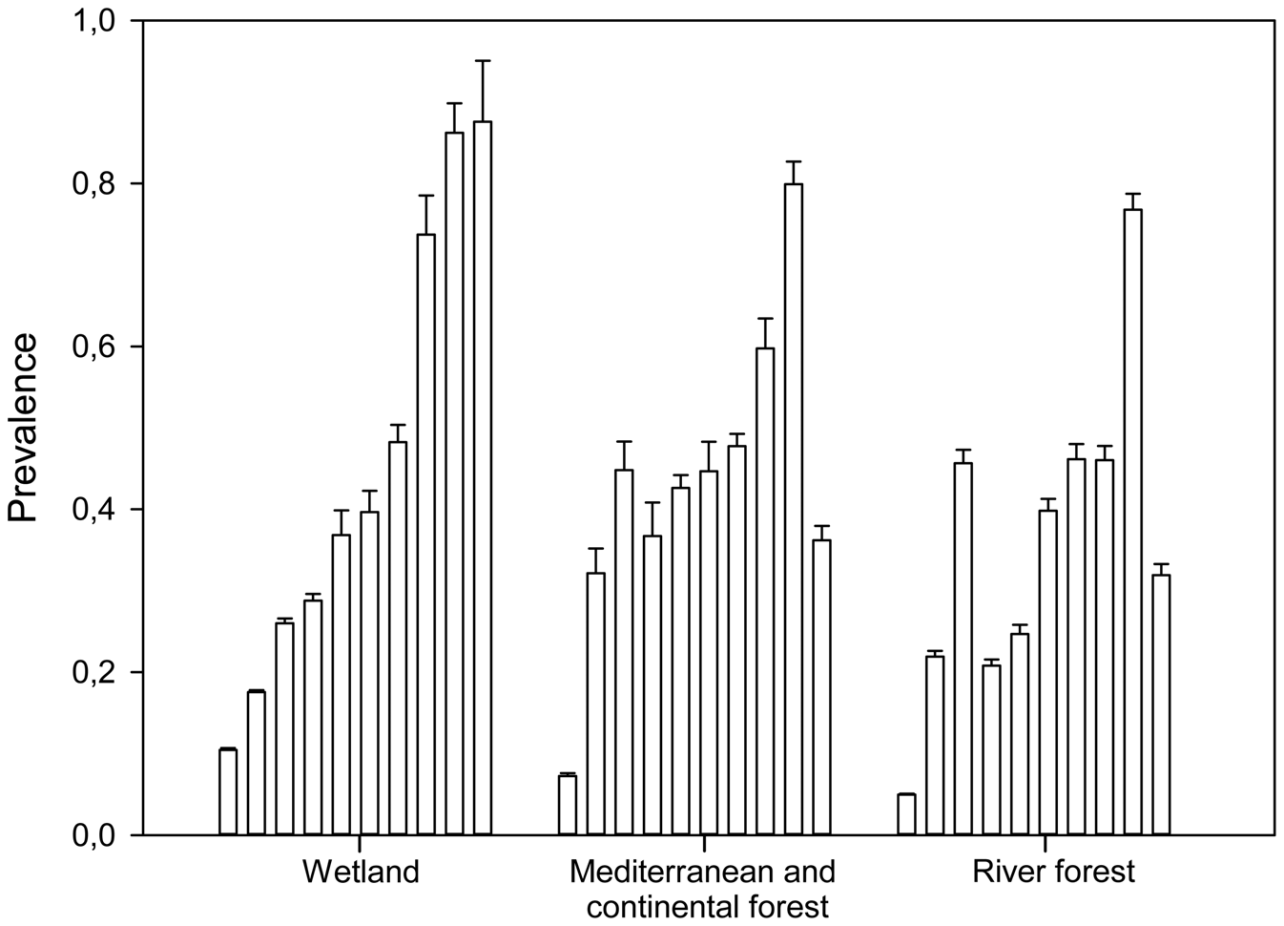
Feather mite prevalence (proportion of birds with feather mites) for ten species of well-sampled resident passerines in three habitats. Species are ordered from left to right within each habitat according to their prevalences in wetlands as follows: *Phylloscopus collybita, Acrocephalus scirpaceus, Carduelis chloris, Cettia cetti, Cyanistes caeruleus, Luscinia megarhynchos, Erithacus rubecula, Serinus serinus, Sylvia atricapilla* and *Fringilla coelebs*. “Breeding” and “habitat” variables were retained as fixed factors in the GLMM, while “observer” and “species” were included as random factors.

Well-sampled resident bird species were quite consistent in their prevalence of mites independently of the habitat where they were sampled (Fig. 4). Repeatability calculated from this subsample led to a lower repeatability estimate (R = 0.255, R_adj_ = 0.324; see Fig. 4 for retained confounding variables).

#### Taxonomic relationships

To explore the distribution of variance in intensity and prevalence across taxonomic levels we ran a GLMM including a nested random effect of species, genus, and family for the whole dataset. Species accounted for 49.7% of total variance on feather mite prevalence, while less was explained by family (21.9%) and very little by genus (0.02%).

### Intensity

**Figure 5:**
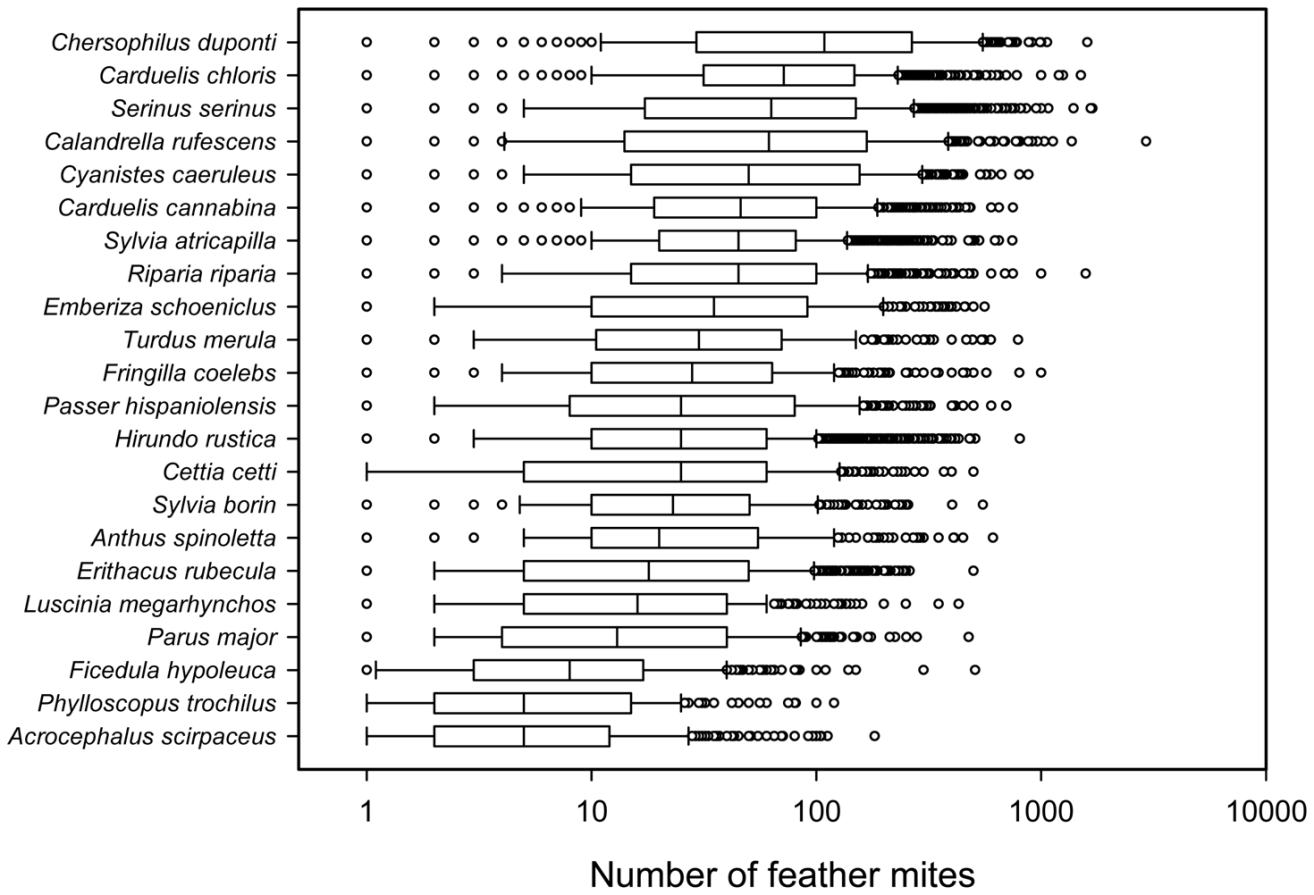
Feather mite intensity of birds. Box-plot of feather mite intensity (on log_10_ axis) for species with more than 300 records. Species are ordered according to their median intensity.

**Figure 6:**
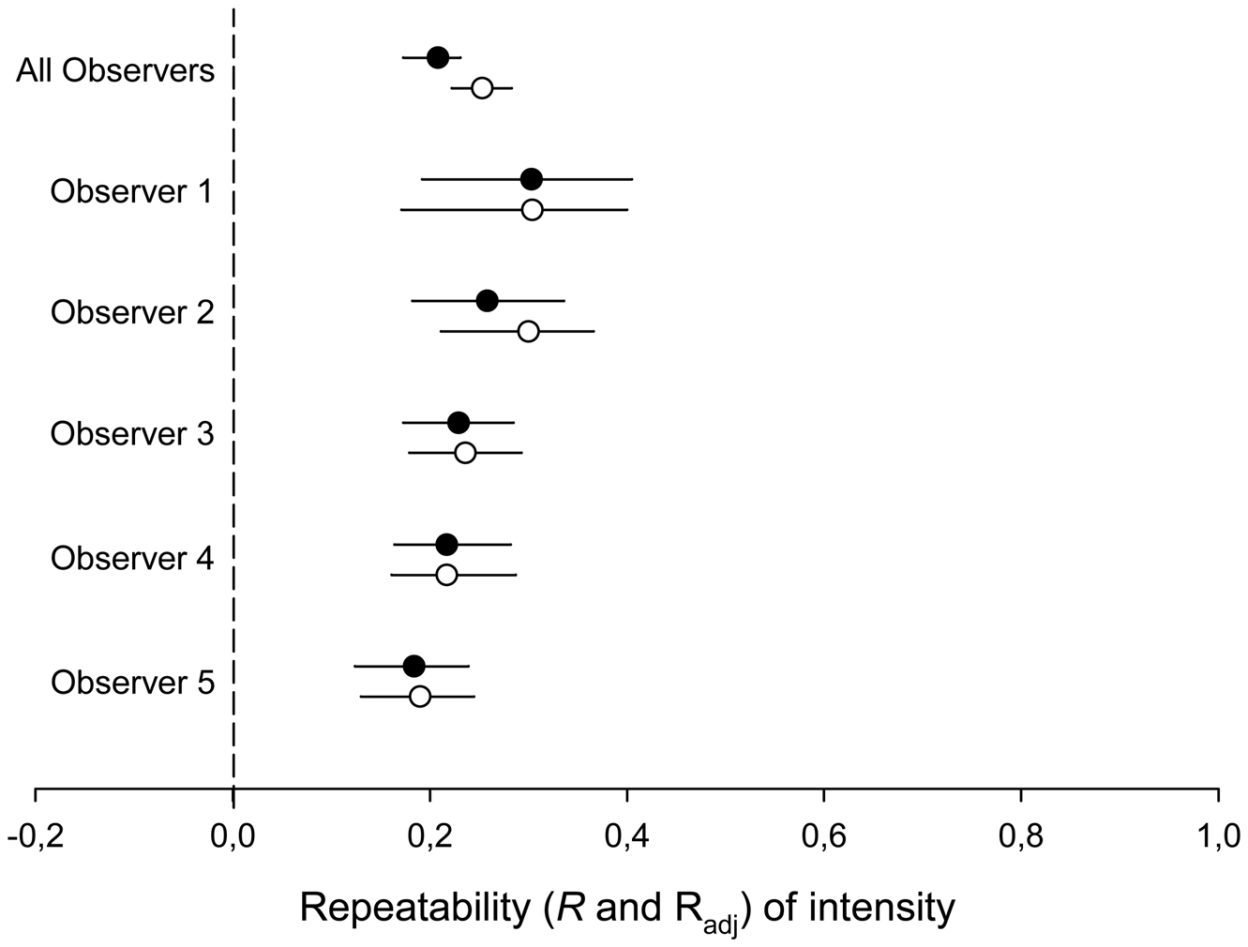
Species repeatability and adjusted repeatability of feather mite intensity. Species repeatability (R; white circles), and adjusted repeatability (R_adj_; black circles) for feather mite intensity with corresponding 95% confidence interval estimates for different intensity data subsets (either the entire dataset, “All observers”, or for data from five different researchers separately). Results are shown separately for five different observers (see Methods), and also for the entire dataset (“All observers”). Adjusted repeatability estimates for observers 1, 3, 4 and 5 were obtained using “breeding” as a fixed effect. For observer 2 “habitat” was retained as a fixed effect. For observers 1 and 2, two and one variables of the autocorrelation term were also added as fixed effects, respectively (see Methods).

Mite intensity varied among individuals by four orders of magnitude (range = 1–10,000 mites), and by two orders of magnitude between species: median range = from 1, e.g. *Oriolus oriolus* and *Petronia petronia*, to 486 mites in *Acrocephalus melanopogon* (Fig. 5; see Table S2 for detailed results for each species). We found a moderate low repeatability for intensity (R = 0.253). Small R could be due to either small between-group (i.e. species) variance or large within-group residual variance (i.e. between individuals of a species). Provided that the between-species variance was quite large, the relatively small R shows that within species variation was also considerable (Fig. 5). Intensity was higher during the non-breeding season and differed among habitats and latitude (Table 2). Moreover, a significant effect of climatic conditions as reflected by PC1 of the PCA analysis was observed, showing that drier and warmer localities held higher feather mite intensities. Controlling for all these confounding variables, the adjusted repeatability was similar to the R estimate (R_adj_ = 0.208, Fig. 6).

**Table 2:** Parameter estimates for LME of the entire dataset of log_10_-intensity of feather mites in birds.

Factor	Description	Estimate	SE	df	t	F	P
Intercept		1.789	0.1269	83	14.09		<.0001
Breeding period				1, 27246		320.72	<.0001
	Non breeding	0.184	0.010	27246	17.88		<.0001
	Breeding	0	.	.	.		.
Habitat				6, 27246		9.86	<.0001
	M. and C. forest	−0.428	0.118	27246	−3.62		0.0003
	Atlantic forest	−0.580	0.119	27246	−4.89		<.0001
	River forest	−0.515	0.119	27246	−4.34		<.0001
	Crops	−0.562	0.118	27246	−4.75		<.0001
	Steppe	−0.555	0.125	27246	−4.43		<.0001
	Wetland	−0.500	0.119	27246	−4.20		<.0001
	Sub. meadow	0	.	.	.		.
							
Climatic conditions	PC1	0.001	<0.001	27246	−5.42	29.41	<.0001
Autocorrelation							
	y	0.078	0.012	27246	−6.37	40.54	<.0001
							

For continuous variables, only statistically significant variables are shown.

#### Different observers

Similar results were found when analysing data separately for five researchers with the greater contribution to the dataset (R range: 0.190–0.304, R_adj_ range: 0.184–0.303; see Fig.6 for the confounding effects retained in the analysis of each subsample).

#### Different habitats

**Figure 7:**
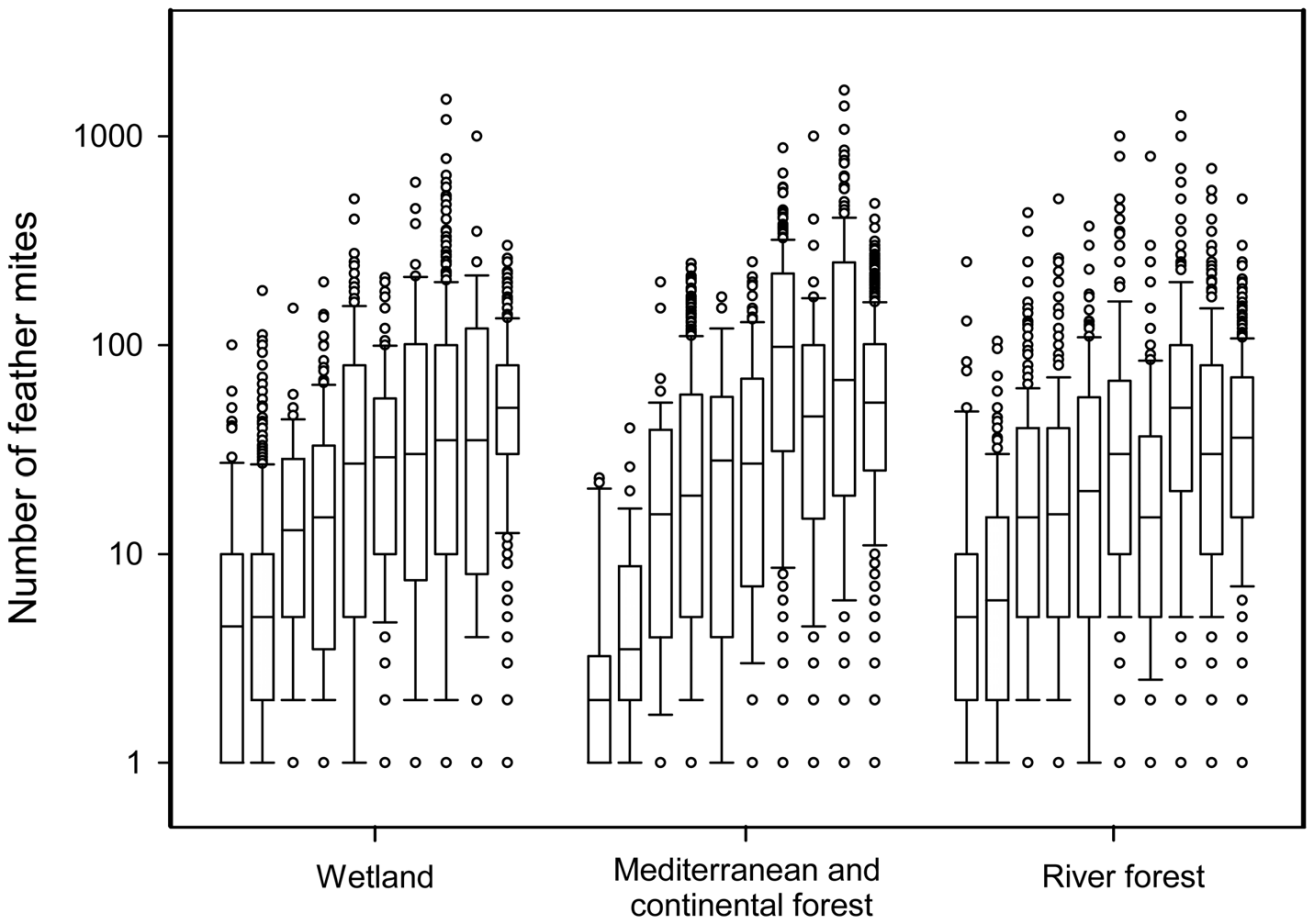
Boxplot of feather mite intensity for ten different species of well-sampled resident passerines in three habitats. Species are ordered from left to right within each habitat according to the intensities of feather mites in individuals captured in wetlands, as follows: *Phylloscopus collybita, Acrocephalus scirpaceus, Luscinia megarhynchos, Erithacus rubecula, Cettia cetti, Fringilla coelebs, Cyanistes caeruleus, Carduelis chloris, Serinus serinus* and *Sylvia atricapilla*. “Breeding”, “habitat” and four variables from the spatial autocorrelation term were retained as fixed factors in LME, while “observer” and “species” were included as random factors.

Again, we analysed a subset of ten well-sampled resident species in three different habitats. Species were consistent in their intensity independently of the habitat where they were found (Fig. 7), leading to R = 0.266 and R_adj_ = 0.226 (see Fig. 7 for retained confounding variables).

#### Taxonomic relationships

Following the scheme for prevalence, we estimated the proportion of total variance among taxonomical levels for feather mite intensity. Species accounted for 20.3% of the variance, while very little was explained by family (0.05%) and nothing by genus (0%).

## Discussion

Despite considerable progress in our understanding of the relationship between host characteristics and feather mite burdens, there was no information on within vs. among bird species variation in prevalence and intensity of mites. Filling this gap is important for knowing at which level (intraspecific or interspecific) future research efforts could be more productive. Feather mite population sizes were highly variable within bird species, leading to considerable overlap between species (e.g. Fig. 5). However, prevalence showed a moderate (R = 0.494) and intensity a lower repeatability (R = 0.208). Interestingly, while several factors potentially biased repeatability estimates, adjusted repeatabilities showed similar results as unadjusted repeatabilities (prevalence: R_adj_ = 0.409; intensity: R_adj_ = 0.253). Overall, thus, bird species consistently differed in their prevalence and intensity of feather mites, while there was a high within-species variance and many species showed similar (average) values. In this regard, differences in prevalence were consistent among species, but high within-species variation was also evident suggesting that analyses at both levels are required. For intensity, however, evidence was much stronger for within-species than for among-species differences. Hence, species-specific approaches could probably be more fruitful for determining causes underlying variation, at least from the host's point of view. Repeatabilities estimated for a sample of researchers were similar, although different variables were retained in each analysis for each observer (likely because researchers differed in species and habitats sampled).

Different reasons could be behind the higher repeatability found for prevalence than for intensity. Prevalence could be more linked to feather mite transmission capacity between habitat islands (bird individuals) of the same island type (host species). For instance, some bird (e.g. breeding sociality) or mite-related species-specific traits (e.g. transmission propensity) could drive the differences in feather mite prevalence between birds. On the other hand, feather mite population size of successfully colonised islands (i.e. intensity) could be more variable within host species because it is potentially affected by different attributes of the colonised island per se. For instance, mite population growth might depend on attributes that are related to the pool of exploitable resources such as bird body condition, [24], [30] or the size of the uropygial gland [3], [48], [49]. Moreover, these mechanisms could be related to environmental variables other than those exerted by the host (e.g. weather conditions hosts do experience, [6], [44], [50]). Interestingly, the first principal component of local climatic conditions was retained only in the model for the intensity, but not for the prevalence of feather mites. This suggests that intensity of feather mite could be partially shaped by environmental factors, while prevalence could be more related with variables shaping the transmission of feather mites such as winter sociality [36]. Otherwise, it is also possible that the differences in repeatability for prevalence and intensity are also influenced by differences in measurement error for both parameters. Although we controlled for systematic bias in intensity and prevalence estimation among observers, we did not control for intra-observer measurement error, which is likely larger when counting the number of feather mites (intensity) than when simply recording whether a bird had or not feather mites (occurrence). In that case, intensity repeatability would be more affected by measurement error than prevalence, leading to lower repeatability estimates for intensity.

Differences among species could be due to variation at higher taxonomic levels. However, we found that species accounted for the highest proportion of the variance, while family and genus accounted for less variance. That shows that intensity and prevalence of feather mites only could be considered a species-specific trait, and not affected by patterns at higher taxonomic levels. Thus, future studies on passerines should be focused on variation in feather mite population in the host at the species level. Moreover, future work including non-passerine species will show whether the pattern found here holds for birds in general.

Ectosymbionts are potentially more affected by environmental conditions than endosymbionts. However, we found small differences in prevalence and intensity of feather mites in bird species among habitats (Figs. 4 and 7). Moreover habitat (for prevalence) and habitat and local climatic conditions (for intensity) played a secondary role compared to the breeding period (Tables 1 and 2, see below). However, climatic conditions seemed to affect the intensity more than habitat. This suggests that while feather mites are highly exposed to external environmental conditions (e.g. humidity, solar radiation), their demography seems more related to bird-mite interactions.

Intensity and prevalence of feather mites were lower during the breeding period, supporting the hypothesis that feather mites are mostly transmitted vertically from parents to offspring by direct physical contact [23], [51]. Hence, our results encourage further studies of the reasons for seasonal changes in feather mite population size, and population dynamics before and after the breeding season. Moreover, we strongly suggest controlling for breeding season in future studies of mite intensity and prevalence as it was the most important variable retained in the statistical models.

Spatial autocorrelation was observed between sampling points in both prevalence and intensity analyses. Interestingly, the latitudinal component of the autocorrelation term was retained in intensity of feather mite models, showing that the number of feather mites was lower at northern latitudes. This pattern deserves further exploration in the future.

Future studies (along the lines initiated by Rózsa [2] and Galván et al. [3]) should continue searching for such species-specific traits of birds that could explain differences in populations of feather mites among bird species (e.g. wing area, uropygial gland size) However, recent approaches to this problem [3] have found that such variables explain a relatively small amount of variance in differences among species, suggesting that complementary approaches are needed. Until now, all attempts (including the present study) have entirely focused on features of bird species. The next approaches will need to incorporate a feather mites' point of view in these analyses (e.g. [34]). A first step would be to test if repeatability of intensity and prevalence of feather mites vary among feather mite species (instead of among bird species), or if repeatability is higher when considering pairs of bird-feather mite species.

## Supporting Information

Table S1 **Intensity of feather mites (Min = Minimum, Max = Maximum, Med = Median) and sample size (N) for each bird species and country.**
(PDF)

Table S2 **Prevalence of feather mites (Prev = Prevalence; i.e. proportion of birds with at least one feather mite; see main text for details) and sample size (N) for each bird species and country.**
(PDF)

Information S1 **Extended ethical statement.**
(PDF)
