# Supplementary material for: Repeatability of Feather Mite Prevalence and Intensity in Passerine Birds
Source: PLoS One. 2014 Sep 12;9(9):e107341. doi: 10.1371/journal.pone.0107341 (PMC4162594; doi:10.1371/journal.pone.0107341)
Supplement: Table S1 — Intensity of feather mites (Min = Minimum, Max = Maximum, Med = Median) and sample size (N) for each bird species and country. (PDF) [file pone.0107341.s001.pdf]

Supporting information 1. Intensity of feather mites (Min=Minimum, Max=Maximum, Med=Median) and sample size (N) for each bird species and country.

	**Denmark**				**Spain**				**Mauritania**				**Morocco**				**Romania**				**Ukraine**				**TOTALS**	
**Bird Species**	**Min**	**Max**	**Med**	**N**	**Min**	**Max**	**Med**	**N**	**Min**	**Max**	**Med**	**N**	**Min**	**Max**	**Med**	**N**	**Min**	**Max**	**Med**	**N**	**Min**	**Max**	**Med**	**N**	**Med**	**N**
Acrocephalus arundinaceus					1	800	30.0	109									4	868	69.0	27					43	136
Acrocephalus melanopogon					1	2128	486.0	62																	486	62
Acrocephalus paludicola					42	150	110.0	3																	110	3
Acrocephalus palustris																	2	128	9.0	11					9	11
Acrocephalus schoenobaenus					1	215	15.0	85									2	64	23.0	38					17	123
Acrocephalus scirpaceus					1	182	5.0	1071									1	92	14.0	12					5	1083
Aegithalos caudatus					1	1199	95.0	165									2	170	40.0	12					78	177
Alaemon alaudipes									5	5	5.0	1													5	1
Alauda arvensis					4	528	25.0	20																	25	20
Anthus berthelotii					1	284	26.0	77																	26	77
Anthus campestris					6	7	6.5	2	155	155	155.0	1													7	3
Anthus pratensis					1	200	43.0	7																	43	7
Anthus spinoletta					1	610	20.0	430																	20	430
Anthus trivialis					5	800	105.0	20									39	465	250.0	9					115	29
Bombycilla garrulus																	31	395	212.5	10					212.5	10
Bucanetes githagineus					1	539	71.0	50																	71	50
Calandrella brachydactyla					1	322	8.5	30																	8.5	30
Calandrella rufescens					1	2928	61.5	372					1	185	43.5	8									61.5	380
Carduelis cannabina					1	750	46.0	979																	46	979
Carduelis carduelis					1	500	15.0	210									2	239	45.5	12					15	222
Carduelis chloris					1	1500	71.5	1739									5	339	68.5	30					71.5	1769
Carduelis citrinella					2	231	44.0	257																	44	257
Carduelis spinus					1	1400	79.5	284																	79.5	284
Cecropis daurica					1	110	29.0	6																	29	6
Certhia brachydactyla					1	217	6.0	16																	6	16
Cettia cetti					1	500	25.0	324																	25	324
Chersophilus duponti					1	1603	107.0	339					5	881	123.0	21									108.5	360
Cisticola juncidis					20	20	20.0	1																	20	1
Coccothraustes coccothraustes					10	246	34.0	6									2	1690	40.0	23					40	29
Cyanistes caeruleus					1	878	50.0	456									2	413	52.5	36					50	492
Cyanopica cyanus					2	60	5.0	15																	5	15
Delichon urbica					1	500	10.0	292									162	238	207.5	6					10	298
Emberiza cia					1	419	20.0	19									240	240	240.0	1					25	20
Emberiza cirlus					1	1100	108.5	33																	108.5	33
Emberiza citrinella					30	120	75.0	2									9	1395	114.0	58					114	60
Emberiza hortulana					50	100	70.0	3									20	155	31.0	5					44	8
Emberiza schoeniclus					1	560	35.0	475									4	420	35.0	7					35	482
Eremopterix nigriceps									1	150	12.0	21													12	21
Erithacus rubecula					1	500	18.0	919									2	243	22.0	25					18	944
Ficedula hypoleuca					1	509	8.0	419									24	24	24.0	1					8	420
Fringilla coelebs					1	1000	25.0	458									4	570	54.0	43					28	501
Fringilla montifringilla					15	40	25.0	3									36	135	68.5	6					47	9
Galerida cristata					1	774	21.0	117	5	5	5.0	1	26	575	122.5	16	8	8	8.0	1					25	135
Galerida theklae					1	587	38.0	78					6	187	17.0	3									37	81

Garrulus glandarius					60	500	69.0	3	8	315	164.0	8					148	11
Hippolais icterina									11	29	20.0	2					20	2
Hippolais polyglotta					1	450	28.0	84									28	84
Hirundo rustica	1	350	27.0	2031	1	804	17.0	1217	1	487	20.0	49	1	300	30.0	986	25	4283
Lanius collurio									1	118	18.0	16					18	16
Lanius excubitor					1	1	1.0	1	61	70	65.5	2					61	3
Lanius meridionalis					15	15	15.0	1									15	1
Lanius senator					6	20	13.0	2									13	2
Locustella fluviatilis									29	248	129.0	6					129	6
Locustella luscinioides					15	500	57.0	14	2	1102	86.0	15					59	29
Locustella naevia					1	500	60.0	120									60	120
Lophophanes cristatus					1	26	13.5	2									13.5	2
Loxia curvirostra					1	545	45.0	171									45	171
Luscinia luscinia									2	769	55.0	39					55	39
Luscinia megarhynchos					1	430	15.0	380	5	136	67.0	9					16	389
Luscinia svecica					1	300	40.0	152									40	152
Melanocorypha calandra					2	731	41.0	18									41	18
Miliaria calandra					1	540	7.5	16	2	89	8.0	3					8	19
Monticola saxatilis					5	5	5.0	2									5	2
Montifringilla nivalis					5	300	30.0	30									30	30
Motacilla alba					1	460	40.0	83	15	111	35.0	8					40	91
Motacilla cinerea					5	139	70.0	7	6	389	61.5	12					69	19
Motacilla flava					2	1200	40.0	88	77	116	96.5	2					40	90
Muscicapa striata					1	140	15.0	18	3	28	8.0	7					9	25
Oenanthe hispanica					7	7	7.0	1									7	1
Oenanthe leucura					40	40	40.0	1									40	1
Oenanthe oenanthe					1	250	10.0	181	2	165	16.0	3					10	184
Oenanthe pleschanka									16	40	31.0	3					31	3
Oriolus oriolus					1	1	1.0	1									1	1
Panurus biarmicus					5	300	30.0	11	1	372	18.0	197					20	208
Parus major					1	476	10.0	283	1	252	27.0	80					13	363
Passer domesticus					1	1605	7.0	252	5	387	52.5	10					8	262
Passer hispaniolensis					1	700	25.0	508	8	35	15.0	6					25	514
Passer montanus					1	472	15.0	195	4	195	43.5	36					20	231
Periparus ater					1	20	4.0	7	4	49	35.0	4					4	11
Petronia petronia					1	1	1.0	1									1	1
Phoenicurus ochruros					1	475	20.0	285	8	97	41.0	4					20	289
Phoenicurus phoenicurus					1	56	20.0	15									20	15
Phylloscopus bonelli					3	18	10.0	3									10	3
Phylloscopus collybita					1	250	4.0	246	1	1	1.0	2					4	248
Phylloscopus sibilatrix									2	32	17.0	2					17	2
Phylloscopus trochilus					1	120	5.0	314	2	27	12.5	8					5	322
Pica pica					3	380	50.0	9	103	160	131.5	2					103	11
Poecile palustris									3	217	20.0	7					20	7
Prunella collaris					1	600	25.0	59									25	59
Prunella modularis					1	1500	20.0	161	26	120	81.0	3					20	164
Pyrrhocorax pyrrhocorax					1	10000	85.0	190									85	190
Pyrrhula pyrrhula					5	15	10.0	3									10	3

Regulus ignicapillus		1	1424	37.0	96			14	14	14.0	1		36	97
Regulus regulus		10	10	10.0	1			7	8	7.5	2		8	3
Remiz pendulinus		1	610	10.0	106			10	163	62.0	5		10	111
Riparia riparia		1	1580	45.0	3524			1	74	27.0	31		45	3555
Saxicola dacotiae		13	720	49.0	3								49	3
Saxicola rubetra		1	40	5.0	21			2	46	22.5	4		5	25
Saxicola torquata		1	175	11.0	178			3	8	5.5	2		11	180
Serinus canaria		9	600	67.5	14								67.5	14
Serinus serinus		1	1689	63.0	1138			11	90	45.5	6		63	1144
Sitta europaea		2	400	22.5	6			2	606	79.0	11		66	17
Sturnus roseus								10	10	10.0	1		10	1
Sturnus unicolor		1	300	15.0	28								15	28
Sturnus vulgaris		1	66	13.5	24			5	292	40.0	9		20	33
Sylvia atricapilla		1	745	45.0	2122			3	279	68.0	75		45	2197
Sylvia borin		1	551	22.0	335			3	93	41.0	22		23	357
Sylvia cantillans		1	2	1.5	2								1.5	2
Sylvia communis		1	17	3.0	19			11	107	61.0	3		5	22
Sylvia curruca								2	173	58.0	17		58	17
Sylvia melanocephala		1	282	5.0	41								5	41
Sylvia nisoria								8	107	57.5	2		57.5	2
Troglodytes troglodytes		1	440	20.0	37			10	46	20.0	5		20	42
Turdus iliacus		26	26	26.0	1								26	1
Turdus merula		1	790	28.0	296			2	543	33.0	57		30	353
Turdus philomelos		1	170	8.0	78			1	118	13.0	5		8	83
Turdus pilaris								10	360	54.0	9		54	9
Turdus torquatus								61	61	61.0	1		61	1
Turdus viscivorus		2	650	50.0	23			144	144	144.0	1		50	24
**TOTAL**	**2031**				**23181**	**24**	**48**				**1187**	**986**		**27457**
