# Supplementary material for: Repeatability of Feather Mite Prevalence and Intensity in Passerine Birds
Source: PLoS One. 2014 Sep 12;9(9):e107341. doi: 10.1371/journal.pone.0107341 (PMC4162594; doi:10.1371/journal.pone.0107341)
Supplement: Table S2 — Prevalence of feather mites (Prev = Prevalence; i.e. proportion of birds with at least one feather mite; see main text for details) and sample size (N) for each bird species and country. (PDF) [file pone.0107341.s002.pdf]

Supporting information 2. Prevalence of feather mites (Prev=Prevalence; i.e. proportion of birds with at least one feather mite; see main text for details) and sample size (N) for each bird species and country.

	**Denmark**		**Spain**		**Kazajstán**		**Mauritania**		**Morocco**		**Romania**		**Ukraine**		**TOTALS**	
**Bird Species**	**Prev**	N	**Prev**	N	**Prev**	N	**Prev**	N	**Prev**	N	**Prev**	N	**Prev**	N	**Prev**	N
Acrocephalus arundinaceus			0.342	486							0.581	43			0.361	529
Acrocephalus melanopogon			0.910	111											0.910	111
Acrocephalus palustris											0.184	49			0.184	49
Acrocephalus schoenobaenus			0.414	295							0.621	58			0.448	353
Acrocephalus scirpaceus			0.183	8432							0.160	50			0.183	8482
Aegithalos caudatus			0.249	787							0.360	25			0.252	812
Alauda arvensis			0.767	30											0.767	30
Anthus berthelotii			0.752	109											0.752	109
Anthus pratensis			0.492	63											0.492	63
Anthus spinoletta			0.913	492											0.913	492
Anthus trivialis			0.846	26							0.667	9			0.800	35
Bucanetes githagineus			0.599	147					0.000	14					0.547	161
Calandrella brachydactyla			0.139	245	1.000	3			0.000	1					0.149	249
Calandrella rufescens			0.853	652	0.667	3			0.522	23					0.841	678
Carduelis cannabina			0.788	1274											0.788	1274
Carduelis carduelis			0.286	969							0.579	19			0.291	988
Carduelis chloris			0.490	3694							0.742	31			0.492	3725
Carduelis citrinella			0.945	272											0.945	272
Carduelis spinus			0.976	292											0.976	292
Cecropis daurica			0.159	44							0.000	2			0.152	46
Certhia brachydactyla			0.051	316											0.051	316
Cettia cetti			0.260	2136											0.260	2136
Chersophilus duponti			0.843	458					0.656	32					0.831	490
Cisticola juncidis			0.436	165											0.436	165
Coccothraustes coccothraustes			0.100	60							0.667	27			0.276	87
Cyanistes caeruleus			0.344	1342							0.609	46			0.353	1388
Cyanopica cyanus			0.294	68											0.294	68
Delichon urbica			0.603	486							1.000	6			0.608	492

Emberiza cia			0.292	65					0.000	1			0.288	66
Emberiza cirlus			0.291	127									0.291	127
Emberiza citrinella			1.000	2					0.879	58			0.883	60
Emberiza schoeniclus			0.558	1192					0.462	13			0.557	1205
Eremopterix nigriceps					0.256	82							0.256	82
Erithacus rubecula			0.482	2257					0.383	47			0.480	2304
Ficedula hypoleuca			0.289	1476					0.200	5			0.288	1481
Fringilla coelebs			0.404	1153					0.773	44			0.418	1197
Galerida cristata			0.660	194	1.000	1	0.548	31	1.000	1			0.648	227
Galerida theklae			0.393	239			1.000	3					0.401	242
Garrulus glandarius			0.111	27					0.500	10			0.216	37
Hippolais pallida			0.027	111									0.027	111
Hippolais polyglotta			0.184	565									0.184	565
Hirundo rustica	0.848	2394	0.306	4031					0.907	54	0.839	1175	0.562	7654
Lanius collurio			0.000	1					0.241	54			0.236	55
Lanius meridionalis			0.080	25									0.080	25
Lanius senator			0.041	73									0.041	73
Locustella luscinioides			0.833	36					0.625	16			0.769	52
Locustella naevia			0.766	167									0.766	167
Lophophanes cristatus			0.020	101									0.020	101
Loxia curvirostra			0.570	300									0.570	300
Luscinia luscinia									0.907	43			0.907	43
Luscinia megarhynchos			0.411	1130					0.250	12			0.409	1142
Luscinia svecica			0.800	421									0.800	421
Miliaria calandra			0.211	76					0.667	3			0.228	79
Montifringilla nivalis			0.652	46									0.652	46
Motacilla alba			0.807	135					0.583	12			0.789	147
Motacilla cinerea			0.632	19					0.417	12			0.548	31
Motacilla flava			0.580	176					1.000	2			0.584	178
Muscicapa striata			0.231	78					0.333	21			0.253	99
Oenanthe oenanthe			0.668	271	0.000	1	0.000	1	1.000	3			0.667	276
Oriolus oriolus			0.033	30					0.000	13			0.023	43
Panurus biarmicus			0.647	17					0.893	215			0.875	232

Parus major	0.187	1654					0.254	177	0.193	1831
Passer domesticus	0.294	1476					0.471	17	0.296	1493
Passer hispaniolensis	0.468	1828	0.800	5			0.455	11	0.469	1844
Passer montanus	0.154	1943					0.547	64	0.166	2007
Periparus ater	0.040	174					0.143	14	0.048	188
Petronia petronia	0.012	81							0.012	81
Phoenicurus ochruros	0.762	390					0.750	4	0.761	394
Phoenicurus phoenicurus	0.133	135			0.000	1			0.132	136
Phylloscopus bonelli	0.048	105							0.048	105
Phylloscopus collybita	0.082	5092					0.035	57	0.082	5149
Phylloscopus trochilus	0.142	2522					0.186	43	0.143	2565
Pica pica	0.172	58					1.000	2	0.200	60
Prunella collaris	0.983	60							0.983	60
Prunella modularis	0.647	255					0.125	8	0.631	263
Pyrrhocorax pyrrhocorax	0.757	984							0.757	984
Regulus ignicapillus	0.513	191					0.000	1	0.510	192
Remiz pendulinus	0.162	1264					0.375	8	0.164	1272
Rhodospiza obsoleta			1.000	26					1.000	26
Riparia riparia	0.777	4543					0.886	35	0.778	4578
Saxicola rubetra	0.189	111					0.400	10	0.207	121
Saxicola torquata	0.369	564					0.200	10	0.366	574
Serinus serinus	0.688	1915					0.000	6	0.686	1921
Sitta europaea	0.102	59					0.636	11	0.186	70
Sturnus unicolor	0.163	178							0.163	178
Sturnus vulgaris	0.688	48					0.500	10	0.655	58
Sylvia atricapilla	0.802	3152					0.907	75	0.804	3227
Sylvia borin	0.438	840					0.688	32	0.447	872
Sylvia cantillans	0.014	148							0.014	148
Sylvia communis	0.059	337					0.064	47	0.060	384
Sylvia curruca							0.219	73	0.219	73
Sylvia hortensis	0.000	34							0.000	34
Sylvia melanocephala	0.149	478							0.149	478
Troglodytes troglodytes	0.184	250					0.375	8	0.190	258

Turdus merula			0.325	1051							0.538	80			0.340	1131
Turdus philomelos			0.282	362							0.211	19			0.278	381
Turdus viscivorus			0.511	47							0.000	1			0.500	48
**Total**	**0.848**	**2394**	**0.416**	**70321**	**0.867**	**37**	**0.314**	**85**	**0.389**	**105**	**0.477**	**1827**	**0.839**	**1175**	**0.430**	**75944**
