# Supplementary material for: Repeatability of Feather Mite Prevalence and Intensity in Passerine Birds
Source: PLoS One. 2014 Sep 12;9(9):e107341. doi: 10.1371/journal.pone.0107341 (PMC4162594; doi:10.1371/journal.pone.0107341)
Supplement: Information S1 — Extended ethical statement. (PDF) [file pone.0107341.s003.pdf]

Supporting Information

Repeatability of feather mite prevalence and intensity in passerine birds
Diaz-Real et al.

Extended Ethical Statement.

O. Frias: Bird ringing was done under the Spanish Ministry of Agriculture, Food and Environment ringing licence number 530340

J. Monros: Bird ringing was done under the Spanish Ministry of Agriculture, Food and Environment ringing licence number 440066.

A. Mestre: Bird ringing was done under the Spanish Ministry of Agriculture, Food and Environment ringing licence number 440102.

F. Atienzar: Bird ringing was done under the Spanish Ministry of Agriculture, Food and Environment ringing licence number 440100.

J. Figuerola: The study was done with the Scientific Permit issued by Generalitat de Catalunya with number 660117.

E. Barba: Bird ringing was done under the Spanish Ministry of Agriculture, Food and Environment ringing licence number 440065.

M. Vogeli, J.L. Tella and D. Serrano: The study was conducted in compliance with the current laws of the Spanish Government and scientific permits issued by the Gobierno de Aragón (permit numbers: R-41504; LCE/mp 24/2007/1254), Gobierno de Navarra (XXX), Junta de Castilla y León (EP/CYL/215/2007) and Junta de Andalucía (SCCFS-AFR/CMM). Bird ringing was done by MV under the Spanish Ministry of Environment ringing licence number 660152.

G. Blanco: Birds were sampled during regular population monitoring activities authorized by the Spanish Ministry of Agriculture, Food and Environment and Regional Governments: ringing license number 530115.

J. Perez Tris: Birds were sampled during regular population monitoring activities authorized by the Spanish Regional Governments to the licensed bird ringers nr. 530326 (JP), 530334 (AB), 530354 (JP-T), 530356 (JAC), 530362 (RM-O), 530365 (JLM), 530370 (CP), 530381 (DP).

A. P. Moller: Bird sampling was done in Denmark under the licence number A351. See documents below for Ukraine licences.

P. L. Pap: Birds were sampled in Romania under the ringing license number is 726965.

C. I. Vágási: Birds were sampled under the ringing license number 423522 emitted by the Romanian Ornithological Center.

JC Senar: Bird ringing was done under the Generalitat de Catalunya ringing licence number SF387

A. Borras: Bird ringing was done under the Generalitat de Catalunya Ringing Licence number SF314

R. Jovani: The study was done with the Scientific Permit issued by Generalitat de Catalunya with number 43430497

«ЗАТВЕРДЖУЮ»
Перший заступник голови
Державного агентства України
з управління зоною відчуження
Д.Г.Бобро
«22» 05 2012 р.

П Р О Г Р А М А

відвідування зони відчуження і зони безумовного (обов'язкового) відселення групою вчених Університету Південної Кароліни та Київського національного Університету
№ 396-і від 21.05.2012 р.

Мета відвідування: проведення спільних досліджень
Маршрут відвідування: № 1, № 2, № 3, № 4, № 6, № 7 (КПП «Дитятки», «Делів», «Прип'ять», «Діброва», «Паришів-2», «Бенівка», «Овруч», «Поліське»)
Обсяг і характер інформації: офіційна загальна інформація про зону відчуження та стан радіоактивного забруднення
Особи, з якими заплановано зустрічі: офіційні особи, що працюють в зоні відчуження
Відповідальний за підготовку та виконання програми: Кірєєв С.І., Полякова М.О. (ДСП «ЧСК»)
Супроводжуюча особа: Чижевський І.В. (ДСП «ЧСК»
Транспорт: автомобілі «Форд Скорпіо» № АІ 7231 ВК (автопричеп № АІ 6785 ХР), «Хюндай Туксон» № АА 7932 ІТ», «Форд Скорпіон» № 73984 КН, «Деу Ланос» № АІ 6972 ВА, «Фіат Добло» № АІ 4898 СХ, «Дачія Логин» № АІ 6436 АО
Термін відвідування: 25 травня – 01 червня 2012 р.

24 травня: **12.00-22.00** Проїзд КПП «Дитятки». Інструктаж з дотримання правил радіаційної безпеки та інформаційна бесіда із представником ДСП «Чорнобильський спецкомбінат»
Прибуття в м. Чорнобиль. Розміщення у будинку іноземного фахівця. (ДП «Чорнобильінтерінформ»
24 травня-01 червня: **05.00- 23.00** Проведення польових досліджень на дослідних полігонах ВКРМРДК «Екоцентр» («Рудий Ліс», «став охолоджувач»), м. Прип'ять (без відвідування будівель), Новошепелицьке лісництво, та в селах зони відчуження (с.с. Буряківка, Красниця, Весняне, Варовичі, Рудня-Іллінецька, Новошепеличі, Копачі, Лелів, Ямпіль, Рудьки, Товстий Ліс, Поліське, Роз'їдже, Паришів, Чистогалівка)
01 червня: **17.00** Від'їзд до м. Києва

Фото- і відеозйомка за маршрутом прямування в межах дії Закону України «Про використання атомної енергії та радіаційну безпеку» та Інструкції «Про порядок охорони державної таємниці, а також іншої інформації...».

Список відвідувачів зони відчуження і зони безумовного (обов'язкового) відселення:

«*ЗАТВЕРДЖУЮ*»
Перший заступник голови
Державного агентства України
з управління зоною відчуження
Д.Г.Бобро
«__22__» ______05______ **2012 р.**

П Р О Г Р А М А

відвідування зони відчуження і зони безумовного (обов'язкового) відселення групою вчених Університету Південної Кароліни та Київського національного Університету
№ 396-і від 21.05.2012 р.

Мета відвідування: проведення спільних досліджень
Маршрут відвідування: № 1, № 2, № 3, № 4, № 6, № 7 (КПП «Дитятки», «Лелів», «Прип'ять», «Діброва», «Паришів-2», «Бенівка», «Овруч», «Поліське»)
Обсяг і характер інформації: офіційна загальна інформація про зону відчуження та стан радіоактивного забруднення
Особи, з якими заплановано зустрічі: офіційні особи, що працюють в зоні відчуження
Відповідальний за підготовку та виконання програми: Кірєєв С.І., Полякова М.О. (ДСП «ЧСК»)
Супроводжуюча особа: Чижевський І.В. (ДСП «ЧСК»
Транспорт: автомобілі «Форд Скорпіо» № АІ 7231 ВК (автопричеп № АІ 6785 ХР), «Хюндай Туксон» № АА 7932 ІТ», «Форд Скорпіон» № 73984 КН, «Деу Ланос» № АІ 6972 ВА, «Фіат Добло» № АІ 4898 СХ, «Дачія Логин» № АІ 6436 АО
Термін відвідування: 25 травня – 01 червня 2012 р.

24 травня: **12.00-22.00** Проїзд КПП «Дитятки». Інструктаж з дотримання правил радіаційної безпеки та інформаційна бесіда із представником ДСП «Чорнобильський спецкомбінат»
Прибуття в м. Чорнобиль. Розміщення у будинку іноземного фахівця. (ДП «Чорнобильінтерінформ»
24 травня-01 червня: **05.00- 23.00** Проведення польових досліджень на дослідних полігонах ВКРМРДК «Екоцентр» («Рудий Ліс», «став охолоджувач»), м. Прип'ять (без відвідування будівель), Новошепелицьке лісництво, та в селах зони відчуження (с.с. Буряківка, Красниця, Весняне, Варовичі, Рудня-Іллінецька, Новошепеличі, Копачі, Лелів, Ямпіль, Рудьки, Товстий Ліс, Поліське, Роз'їдже, Паришів, Чистогалівка)
01 червня: **17.00** Від'їзд до м. Києва

Фото- і відеозйомка за маршрутом прямування в межах дії Закону України «Про використання атомної енергії та радіаційну безпеку» та Інструкції «Про порядок охорони державної таємниці, а також іншої інформації...».

Список відвідувачів зони відчуження і зони безумовного (обов'язкового) відселення:

Додаток до Програми № 396-і від 21.05.2012 р.

Список

обладнання, що ввозиться до зони відчуження і зони безумовного (обов'язкового) відселення для виконання досліджень

на період з 24 травня - 1 червня 2012р.

- Каністра металева 10 л - 1 шт.
- Центрифуга - 1 шт.
- Переносна електростанція SDMO - 1 шт.
- Каністра металева 5 л - 1 шт.
- Кондиціонер мобільний SATURN - 1 комплект.
- Палатка-тент Royal House каркасна - 3 шт.
- Стіл складний з лавами - 2 комплекти.
- Жердина стальна хромована - 108 шт.
- Цегла із свинцю - 10 шт.
- Жердина алюмінієва - 12 шт.
- Пилка ручна - 2 шт.
- Мачете - 2 шт.
- Драбина металева складна - 1 шт.
- Чайник електричний - 1 шт.
- Подовжувач 220В, 50 м - 1 шт.

«ПОГОДЖЕНО»

Завідувач сектору з питань режиму

_________________ **С. В. Бірук**

«21» ___05___ 2012 р.

«ЗАТВЕРДЖУЮ»
Перший заступник голови
Державного агентства України
з управління зоною відчуження
Д.Г.Бобро
«_17_» _06_ 2012 р.

П Р О Г Р А М А

відвідування зони відчуження і зони безумовного (обов'язкового) відселення
групою вчених Університету Південної Кароліни
та Київського національного Університету
№ 560-і від 19.06.2012 р.

Мета відвідування: проведення спільних досліджень
Маршрут відвідування: № 1, № 2, № 3, № 4, № 6, № 7 (КПП «Дитятки», «Лелів», «Прип'ять», «Діброва», «Овруч», «Поліське»)
Обсяг і характер інформації: офіційна загальна інформація про зону відчуження та стан радіоактивного забруднення
Особи, з якими заплановано зустрічі: офіційні особи, що працюють в зоні відчуження
Відповідальний за підготовку та виконання програми: Кірєєв С.І., Полякова М.О. (ДСП «ЧСК»)
Супроводжуюча особа: Чижевський І.В. (ДСП «ЧСК»
Транспорт: автомобілі «Форд Скорпіо» № АІ 7231 ВК з автопричепом № АІ 6785 ХР (водій Міліневський Леонід, , «Хюндай Туксон» № АА 7932 ІТ, «Форд Скорпіон» № 73984 КН (водій Міліневський Геннадій), «Деу Ланос» № АІ 6972 ВА (водій Міліневський Сергій), «Фіат Добло» № АІ 4898 СХ (Іваненко Максим), «Дачія Логин» № АІ 6436 АО (водій Чижевський Ігор)
Термін відвідування: 22 червня - 02 липня 2012 р.

22 червня: **15.00-22.00** Проїзд КПП «Дитятки». Інструктаж з дотримання правил радіаційної безпеки та інформаційна бесіда із представником ДСП «Чорнобильський спецкомбінат»
Прибуття в м. Чорнобиль. Розміщення у будинку іноземного фахівця. (ДП «Чорнобильінтерінформ»
22 червня-02 липня: **05.00- 23.00** Проведення польових досліджень на дослідних полігонах ВКРМРДК «Екоцентр» («Рудий Ліс», «став охолоджувач»), м. Прип'ять (без відвідування будівель), Новошепелицьке лісництво та в селах зони відчуження (с.с. Весняне, Рудня-Іллінецька, Новошепеличі, Поліське, Чистогалівка)
02 липня: **17.00** Від`їзд до м. Києва

Фото- і відеозйомка за маршрутом прямування в межах дії Закону України «Про використання атомної енергії та радіаційну безпеку» та Інструкції «Про порядок охорони державної таємниці, а також іншої інформації...».

Список відвідувачів зони відчуження і зони безумовного (обов'язкового) відселення:

№№	Країна	Прізвище та ім'я	Дата народження	№ паспорта
1.	США	Тимоті Мюссе	10.10.1958	450066018
2.	Італія	Андреа Бонісолі-Алкуаті	02.07.1980	YA0427616
3.	Україна	Станіслав Рушковський	01.01.1972	ЕС643677
4.	Україна	Леонід Міліневський	09.11.1946	СК079746
5.	Україна	Геннадій Міліневський	30.04.1951	СН852438
6.	Україна	Андрій Рожок	14.04.1979	МЕ 370281
7.	Україна	Сергій Міліневський	08.04.1971	СК409357
8.	Україна	Євген Тукаленко	05.07.1978	СН 438442
9.	Україна	Максим Іваненко	18.03.1968	СО760042
10.	Україна	Володимир Безруков	23.08.1952	СН 527047

Загальна кількість відвідувачів: 10 осіб

Фото- і відеозйомка за маршрутом прямування в межах дії Закону України «Про використання ядерної енергії та радіаційну безпеку» та Інструкції «Про порядок охорони державної таємниці, а також іншої інформації...».

Директор **М.В.Бойко**

Центру ОТЗ ДСП «ЧСК»

«ПОГОДЖЕНО»

Завідувач сектора з питань режиму

С.В.Бірук

« 13 » 06 2012 р.

Начальник режимно-секретного відділу ДСП «ЧСК»

С.В.Манжос

« » 06 2012 р.

Підстава: Запит № 1573 від 13.06.2012 р.

№№	Країна	Прізвище та ім'я	Дата народження	№ паспорта
1.	США	Тимоті Мюссе	10.10.1958	450066018
2.	Італія	Андреа Бонісолі-Алкуаті	02.07.1980	YA0427616
3.	Данія	Андерс Моллер	26.12.1953	200080477
4.	Данія	Вільям Карое Аарестрап	27.08.1932	200569468
5.	Україна	Геннадій Міліневський	30.04.1951	СН852438
6.	Україна	Леонід Міліневський	09.11.1946	СК079746
7.	Україна	Сергій Міліневський	08.04.1971	СК409357
8.	Україна	Станіслав Рушковський	01.01.1972	ЕС643677
9.	Україна	Максим Іваненко	18.03.1968	СО760042
10.	Україна	Придюк Микола	19.11.1968	СО286687

Загальна кількість відвідувачів: 10 осіб

Фото- і відеозйомка за маршрутом прямування в межах дії Закону України «Про використання ядерної енергії та радіаційну безпеку» та Інструкції «Про порядок охорони державної таємниці, а також іншої інформації...».

Заступник директора
Центру ОТЗ ДСП «ЧСК» **В.П.Махно**

«ПОГОДЖЕНО»
Завідувач сектора з питань режиму
С.В.Бірук
«21» ________________ 2012 р.

Начальник режимно-секретного відділу ДСП «ЧСК»
С.В.Манжос
«____» ________________ 2012 р.

Режимно-секретний відділ

Підстава: Запит № 1177 від 14.05.2012 р.
